# Patient-Reported Outcomes Following Total Knee Replacement in Patients <65 Years of Age—A Systematic Review and Meta-Analysis

**DOI:** 10.3390/jcm9103150

**Published:** 2020-09-29

**Authors:** Jason Trieu, Daniel J. Gould, Chris Schilling, Tim Spelman, Michelle M. Dowsey, Peter F. Choong

**Affiliations:** 1 Department of Surgery, University of Melbourne, Level 2, Clinical Sciences Building, 29 Regent Street, Fitzroy 3065, Australia; d.gould@student.unimelb.edu.au (D.J.G.); chris.schilling@unimelb.edu.au (C.S.); tim@burnet.edu.au (T.S.); mmdowsey@unimelb.edu.au (M.M.D.); pchoong@unimelb.edu.au (P.F.C.); 2 Department of Orthopaedic Surgery, St Vincent’s Hospital 41 Victoria Parade, Fitzroy 3065, Australia

**Keywords:** patient-reported outcomes, total knee replacement, total knee arthroplasty, osteoarthritis, middle-aged

## Abstract

An increasing number of total knee replacements (TKRs) are being performed in response to the growing burden of osteoarthritis. Patients <65 years of age represent the fastest growing group of TKR recipients and are expected to account for an increasing number of primary and revision procedures. Concerns have been raised about the outcomes that can be expected by this age demographic who are more active, physically demanding, and have longer life expectancies compared to older TKR recipients. This systematic review and meta-analysis evaluated the effectiveness of TKR for osteoarthritis in patients <65 years of age, compared to older individuals. A systematic search of Embase and Medline was conducted to identify studies which examined patient-reported outcomes measured using disease-specific and generic health-related quality of life instruments. Ten studies met our inclusion criteria and were included in this review. These studies comprised 1747 TKRs performed between 1977 and 2014. In the meta-analysis of two prospective studies (288 TKRs), patients <65 years of age were able to attain large and clinically meaningful improvements in pain, function, and quality of life. One of these studies (61 TKRs) suggested that patients <55 years of age attained a larger degree of improvement compared to older individuals. Results into the second postoperative decade were less certain, with some data suggesting a high prevalence of pain and patterns of functional decline. Further research is required to investigate longer-term outcomes following TKR for osteoarthritis in younger patients.

## 1. Introduction

Total knee replacement (TKR) remains a successful and effective procedure in the treatment of knee osteoarthritis [1]. As the osteoarthritis burden grows, an increasing number of TKRs are being performed; high-volume countries like the United States are projecting nearly 3.5 million procedures for the year 2030 [2]. Comparable rates of growth are similarly projected for countries with high rates of TKR utilisation per capita [3]. Australia is anticipated to perform up to 161,000 TKRs in 2030, and the United Kingdom up to 1.2 million TKRs in 2035 [4,5] Growing patient demand, expectations for improved quality of life, and increasing implant survivorship have contributed to the expansion of TKR towards a greater number of younger patients [6]. In particular, patients <65 years of age represent the fastest growing population of TKR recipients and are expected to account for more than 50% of knee replacement procedures by the year 2030 [3,7].

The rapidly expanding use of TKR by younger patients presents a number of different challenges. Due to more active lifestyles, greater physical demands, and longer lifespans compared to traditionally older recipients of TKR, concerns have been raised about the higher rates of revision surgery faced by this group [8]. Bayliss et al. have reported an increased lifetime risk of revision of up to 35% in male patients who undergo TKR in their early 50s [9]. Furthermore, the excellent pain, function, and quality of life outcomes reported in the literature have mostly related to older and less active patient populations, and therefore may not translate to younger patients [8]. Given these complex considerations, the decision to perform TKR in younger patients should be fully informed by an understanding of the risk-benefit profile of the procedure, ensuring that this procedure is able to meet patient expectations (performance and longevity) in terms of what can be realistically achieved through surgery.

Understanding the expected outcomes that can be achieved with TKR in this younger demographic will be critically important to assist with the management of patient expectations and provide guidance for the rapidly growing use of TKR in this cohort [10]. Existing reviews have examined TKR outcomes using the Knee Society Score (KSS); whilst not strictly a patient-reported outcome measure due to the inclusion of clinician-based assessment, it does contain within it a patient-reported component, and remains one of the most widely used instruments in clinical practice [11,12]. Furthermore, outcomes have been evaluated across a heterogenous mix of diagnoses which include both inflammatory arthritis and osteoarthritis; outcomes for inflammatory arthritis are not directly comparable to those for osteoarthritis which remains the most common indication for TKR [1,13,14]. In comparison, the focus has shifted towards the use of patient-reported outcome measures and the increasing use of TKR in patients aged <65 years with a diagnosis of osteoarthritis [15]. To address these limitations, we sought to: (1) synthesise the current evidence for the patient-reported pain, function, quality of life, and satisfaction that can be expected for this age demographic, compared to older individuals, (2) provide a clinical interpretation of the changes in instrument scores, and (3) evaluate longer-term results to provide realistic expectations for patients and clinicians and guide the expanding use of TKR in this cohort.

## 2. Material and Methods

This systematic review was conducted in accordance with Preferred Reporting Items for Systematic Reviews and Meta-Analyses (PRISMA) guidelines [16].

### 2.1. Search Strategy

The search strategy was developed using the Boolean operators “AND” and “OR”, and “total knee arthroplast *.ti”, “total knee arthroplast *.ab”, “total knee arthroplast *.ti”, “total knee arthroplast *.ab”, “outcome *.ti”, “outcome *.ab”, “arthroplasty, replacement, knee”, “total knee arthroplasty”, “middle aged”, and “treatment outcome”. The strategy on Embase combined ((total knee replacement or total knee arthroplast *) and outcome *).ti. OR ((total knee replacement or total knee arthroplast *) and outcome *).ab. On Medline, Medical Subject Headings (MeSH) terms “arthroplasty, replacement, knee” OR “total knee arthroplasty” AND “middle aged” AND “treatment outcome” were combined. Searches were limited to full text, English language, and publication date from 2004. Backwards citating chaining, Cochrane databases, and Google Scholar were searched using the same question themes to identify additional articles for screening.

### 2.2. Inclusion and Exclusion Criteria

The following inclusion criteria were determined a priori and applied to study selection: (1) patient age <65 years in the study cohort or a subgroup within the study cohort, (2) osteoarthritis as the indication for surgery in ≥90% of cases, (3) primary TKR as the treatment, (4) disease-specific or health-related quality of life instrument score as an outcome measure, and (5) minimum follow-up of 6 months. Exclusions were: (1) non-English language publications, (2) publication year prior to 2004, (3) grey literature, (4) systematic reviews. English language abstracts of non-English publications were screened where available; no studies failed to meet inclusion for full text screening as a result of publication in a language other than English.

### 2.3. Study Selection

**Figure 1:**
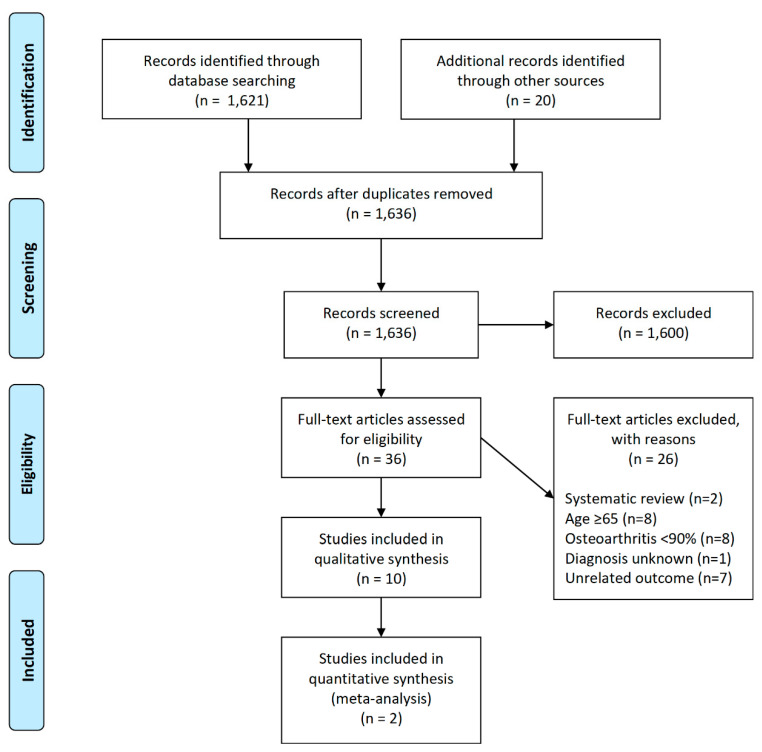
Preferred Reporting Items for Systematic Reviews and Meta-Analyses (PRISMA) flow diagram of search algorithm.

Following the search, references were exported to EndNote X9 for screening. One review author (JT) performed initial title screening and excluded articles not considered relevant to the topic of investigation. Two review authors (JT and DG) then performed title, abstract, and full-text screening. Eligible articles were included for final review following discussion and consensus of two review authors (JT and DG). Reasons for exclusion following full-text screening are illustrated in the PRISMA flow diagram (Figure 1).

### 2.4. Data Extraction

A data template was developed in Microsoft Excel 2016 (Microsoft, Redmond, WA, USA). The following data were extracted: (1) study design, (2) patient demographics, (3) surgery characteristics, (4) outcome measures including duration of follow-up, (5) satisfaction. For studies that reported results according to age subgroups, only the results relevant to our age subgroup were extracted. Corresponding authors were sent an email requesting further information for data that required clarification.

### 2.5. Statistical Analysis

Differences exist across disease-specific patient-reported outcome measures (PROMs) both in terms of the range of the scoring scale, and the direction of the scale (whether a higher score represents a better or worse outcome). To facilitate ease of comparison, we standardised all PROMs to a range of 0 to 100. For measures where a higher score originally represented a worse outcome, this was reversed so that a higher score represented a better outcome [17]. Due to variation in the reporting of data, only a limited meta-analysis of two prospective studies was performed because key data were unavailable (missing baseline scores, unreported standard deviations). Where studies reported standard errors, these were used to calculate standard deviations in accordance with Cochrane guidelines [18]. Meta-analysis was performed using a random effects model, which assumes that true intervention effects may vary across studies. The random effects model was chosen over the fixed effect model due to methodological heterogeneity related to study design, reporting of patient and procedural characteristics, and follow-up duration that was encountered during full text screening and data abstraction [19]. For the pain and function subscales of disease-specific instruments, and physical and mental health subscales for generic health-related quality of life instruments, analysis was reported using standardised mean differences. No individual studies contributed more than one patient-reported outcome measure to each subscale that was analysed. Heterogeneity was assessed with Cochran’s Q and considered present if *p* < 0.05; the percentage of heterogeneity not due to chance was assessed using I^2^, and ≥ 50% was considered substantial [18]. Statistical analysis was performed in R version 3.5.3 (R Core Team, R Foundation for Statistical Computing, Vienna, Austria) using package “meta” [20]. 

### 2.6. Assessment of Methodological Quality

Two review authors (JT and DG) independently performed the qualitative risk of bias evaluation for each study using a modification of Cochrane’s Risk of Bias in Non-Randomized Studies—of Interventions (ROBINS-I) tool [21]. ROBINS-I assesses six domains: confounding, selection of participants, classification of interventions, missing data, outcome measurement, and selective reporting [22]. Disagreements were resolved by consensus from both review authors (JT and DG). There were no disagreements which required involvement of a third review author.

### 2.7. Clinically Meaningful Improvements

The minimal clinically important difference (MCID) is commonly used to assess whether a change in an instrument score represents a clinically meaningful change for the patient. This represents the difference in scores between patient groups that perceive a minimal but clinically meaningful difference and patient groups who perceive no difference [23,24]. The MCID is typically calculated using either the anchor or distribution methods [25]. The reference method for each study is presented in Table 1. Changes in scores across different instruments which measure the same underlying construct are compared using the standardised mean difference (SMD). SMDs are measured in units of the pooled standard deviation of the change in scores, and therefore do not have a defined scale. An effect size of 0.2 SMDs is considered small, 0.5 SMDs considered medium, and 0.8 SMDs considered large [26]. 

## 3. Results

### 3.1. Search Strategy

A total of 1641 studies were identified through the search strategy. There were 1636 remaining after removal of 5 duplicates. Following title and abstract screening, 36 proceeded to full-text screening, and 26 were excluded from the review, with the reasons for their exclusion stated. A total of 10 studies were eligible for inclusion. The PRISMA flow diagram illustrates the results of the search (Figure 1).

### 3.2. Study, Patient, and Surgery Characteristics

Six studies were prospective, and four were retrospective (Table 2). A total of 1747 TKRs were performed between 1977 and 2014. Follow-up exceeded 90% for five of the six prospective studies. Cohorts ranged from 37 to 673 TKRs, with five studies each reporting ≤100 TKRs. The overall rate of osteoarthritis was 96.1%. Mean or median age at time of surgery was 45 to 58 years, and the gender distribution was 36.6% male and 63.4% female. Eight studies reported data on prosthesis constraint: cruciate-retaining prostheses were used in 43.8% of cases (623 of 1423 TKRs) and posterior-stabilised prostheses in 56.2% of cases (800 of 1423 TKRs). Six studies reported data on patellar resurfacing: the patellar resurfacing rate was 68.9% (821 of 1192 TKRs).

### 3.3. Disease-Specific Instruments

**Figure 2:**
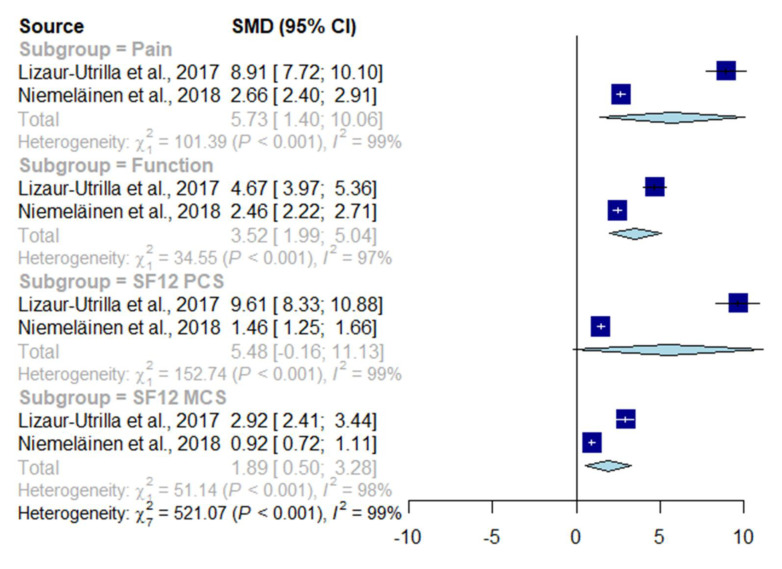
Forest plot of change in patient-reported pain, function, and quality of life (physical health and mental health) following primary total knee replacement (TKR), assessed using standardised mean differences.

Ten studies reported results for at least one disease-specific instrument (Table 1) [33,34,35,36,37,38,39,40,41]. With the exception of one study, all studies reported improvements exceeding the reference MCID where available (Table 1) [35]. Mean or median follow-up periods ranged from 6 months to 25.1 years. Two prospective studies (288 TKRs, mean or median follow-up of 2 years to 12 years) contributed to the meta-analysis [33,42]. The pooled effect size for improvement in pain was 5.73 SMDs (95% CI 1.40 to 10.06) and for function was 3.52 SMDs (95% CI 1.99 to 5.04) (Figure 2). Heterogeneity was substantial for pain (I^2^ = 99%) and function (I^2^ = 97%).

### 3.4. Generic Health-Related Quality of Life Instruments

Four studies reported the PCS and MCS for a health-related quality of life instrument; none reported a utility score (Table 1) [33,34,38,42]. Improvements exceeded the reference MCID where available (Table 1). Mean or median follow-up ranged from 1 to 12 years (Figure 2). Two prospective studies (288 TKRs, mean or median follow-up of 2 years to 12 years) contributed to the meta-analysis [33,42] The pooled effect size for improvement in the PCS was 5.48 SMDs (95% CI −0.16 to 11.13), and in the MCS was 1.89 SMDs (95% CI 0.50 to 3.28) (Figure 2). Heterogeneity was substantial for the PCS (I^2^ = 99%) and MCS (I^2^ = 98%).

### 3.5. Satisfaction

Satisfaction was reported by four studies (Table 3) [33,34,36,42]. Measurement instruments included a four-point Likert scale and a Visual Analogue Scale [34,42]. Up to 84% of patients reported being satisfied or very satisfied with the outcome 2 years following surgery. This ranged from 72.4% for recreation, to 80.1% for work, and 85.1% for pain. In this patient cohort, 98% considered undergoing surgery again, and 96% would recommend the surgery to others. Two studies reported satisfaction ranging from 90.2% to 93.5% at up to 10 years following surgery; in these studies, the instrument was not defined [33,36].

### 3.6. Methodological Quality

The risk of bias assessment relates to our confidence that the degree of improvement in patient outcomes can be attributed to TKR, and not to differences in patient or procedural characteristics. The confounding domain demonstrated the highest risk of bias across studies (Table 4). This was primarily related to retrospective study designs, small patient cohorts, and limited reporting of characteristics associated with patient outcomes following knee replacement surgery. The risk of bias across other domains was generally due to a combination of issues relating to missing pre-operative instrument scores, inconsistencies with the reporting and classification of surgical characteristics, and insufficient detail describing missing data. In four studies, TKRs were performed by a single surgeon [33,35,41,43]. A summary of the methodological quality assessment for each study is provided in Table 4.

## 4. Discussion

The findings of this review suggest that younger patients attain clinically meaningful improvements exceeding MCIDs across patient-reported pain, function, and quality of life following TKR for osteoarthritis. Satisfaction in this cohort was equivalent to the results achieved in the broader TKR literature. The degree of improvement was considered large (pooled effect size >0.8 SMDs) across pain, function, and quality of life. However, the limited data available for analysis resulted in a high degree of uncertainty around estimates, particularly for the PCS. Some studies suggest a high prevalence of pain and patterns of functional decline in the second post-operative decade, and residual dissatisfaction in a percentage of patients remains an issue [33,38,40]. Limited evidence suggests that improvements observed in younger individuals is generally equivalent and potentially greater than those attained by older individuals [33,34,35,38].

The greatest improvement was reported in the pain subscale (effect size >0.8 SMDs) over a median follow-up period of up to 12 years. Large improvements were also attained in the function subscale. Studies which did not contribute to the meta-analysis reported comparable post-operative scores or improvements over follow-up periods ranging from 6.5 to 16.8 years for patients <55 years of age [36,39]. Scores were available for two instruments which are not strictly patient-reported outcome measures, although they each have a patient-reported component—the KSS, and the Hospital for Special Surgery (HSS) knee score. Improvements in the KSS exceeded the MCID; for the HSS, the MCID has not been established [33,36,37,39,41]. These results support the effectiveness of TKR in relieving pain and improving function for patients <65 years of age with osteoarthritis.

The greatest improvement to quality of life was in physical health, with potentially large improvements over a median follow-up period of up to 12 years. However, there was a high degree of uncertainty around estimates (95% CI of −0.16 to 11.13). Large improvements were reported in mental health over the same period of follow-up. Improvements in quality of life were equivalent to those achieved by older patients. In one study (227 TKRs), 98% considered undergoing the procedure again and 96% would have recommended the procedure to others [42]. Satisfaction of 84% at 2 years post-operatively was equivalent to that in older patients, and consistent with the broader literature [44]. Despite satisfaction rates that are consistent with the TKR literature, satisfaction remains a complex area influenced by a range of factors [45]. The discordance between satisfaction with TKR versus the higher percentage that would undergo or recommend the procedure suggests that some expectations remain unmet [46]. This in particular reinforces the need for treating clinicians to establish clear expectations around the present uncertainties of longer-term results.

The performance of TKR into the second decade following surgery is less predictable. The decision to proceed with TKR, compared to alternative joint-preserving strategies, should be carefully weighed against the elevated risk of revision surgery in this cohort. With younger patients having longer life expectancies and facing higher lifetime rates of revision surgery, the longer-term results will be an important consideration in the decision to undergo TKR. In addition to one of the studies included in the meta-analysis, four other studies had mean or median follow-up periods exceeding ten years, two of which did not report outcomes using patient-reported instruments [33,37,39,40]. For patient-reported outcomes, improvement in the Western Ontario and McMaster Universities Osteoarthritis Index (WOMAC) at a mean of 16.8 years was comparable to improvements reported by studies that were included in the meta-analysis, and another study reported only the post-operative score for the Oxford Knee Score (OKS) and so the degree of improvement could not be determined [39,40]. An interesting finding was the high prevalence of pain at a mean follow-up of 15.5 years, with 41% of the unrevised TKRs in this cohort reporting moderate or severe pain according to the OKS pain subscale [40]. A trend identified across both younger and older patients was the increasing functional impairment with advancing age, which the authors suggest may be related to an increasing comorbidity burden and declining activity levels [33,38]. Uncertainty of longer-term outcomes, a high prevalence of pain, and patterns of functional decline are concerning and should be examined.

### 4.1. Implications for Practice and Research

The success of TKR has seen a rapid growth in its utilisation across a number of countries, and also its expansion into younger age groups [3,15]. In response, health services have shown increasing interest in the use of quality metrics including patient-reported outcome measures to evaluate the impact and value of surgery [10,47,48]. These instruments reflect patient-relevant outcomes including pain, function, and quality of life which are some of the primary indications for TKR that are not captured by traditional metrics such as prosthesis survival [40]. With increasing prosthesis longevity reported by national registries, younger individuals will be expected to live with a prosthesis for a longer period of time, and longer-term patient outcomes therefore become an important consideration in the decision to undergo TKR [49]. Although the limited data available support the appropriate use of TKR in this younger cohort, there remain a number of concerns which require further investigation.

Whilst TKR provides clinically meaningful improvements to pain, function, and quality of life in the first decade, a large degree of uncertainty surrounds outcomes and expectations beyond this period. There is a greater likelihood that outcomes may deteriorate whilst the risk of revision surgery increases. Our most comprehensive understanding of prosthesis survivorship currently stems from established national joint registries, with cumulative 15-year revision rates ranging from 4.3% to 15.5% reported by the Australian Orthopaedic Association’s National Joint Replacement Registry [49]. However, younger patients are expected to use their prosthesis beyond the period for which data are currently available. Bayliss et al. recently investigated the lifetime risk of revision surgery, and of concern is the marked increase in lifetime revision rate for TKRs from approximately 15% for those undergoing surgery between ages 60–70, rising a few percentage points for females undergoing surgery at age 50–60, but alarmingly more than doubling to 35.0% for males undergoing surgery at age 50–54 [9]. These data lend support to concerns raised by other authors about the rising use of TKR in younger patients, where data and certainty of outcomes are relatively lacking in comparison to older patients and hence appreciation of longer-term consequences may not be adequately informed [8]. We suggest that due consideration should be provided to alternative strategies that can address symptoms and potentially delay the need for arthroplasty until later stages where outcomes are more predictable and the lifetime risk of revision is lowered. A role for the selective use of joint-sparing techniques such as high-tibial osteotomy in earlier stages of disease progression can be demonstrated if they are able to deliver improved patient outcomes or reduce the rate of revision surgery [50]. Despite potentially higher costs with staged procedures to delay the need for TKR, this option may remain cost-effective if the revision risk can be mitigated.

The investigation of these longer-term outcomes will be required to help inform patients about the realistic results that can be expected. Importantly, patients for whom the expected outcomes of TKR do not align with their expectations may be redirected to alternative and more appropriate treatment strategies. The focus of investigation should now shift towards strategies aimed at maintaining the benefit of TKR throughout the longer-term and minimising dissatisfaction following surgery. Research suggests that there is a strong role for the identification of long-term pain, function, and quality of life trajectories following TKR, where strategies targeting the modifiable predictors of poor response to surgery may have the potential to improve longer-term patient-reported outcomes [51,52]. Furthermore, clinical joint replacement registries have been highly effective in monitoring the long-term survivorship of prostheses to inform practice, and are similarly well placed to facilitate the systematic collection and monitoring of quality metrics including patient-reported outcomes over longer periods of sustained follow-up [10,47,53,54]. 

Greater emphasis should be placed on the consistent use and reporting of validated instruments. Reporting of pre-operative baseline scores will enable comparison of outcomes, and consistent reporting of data to include means with standard deviations should be adopted. Where feasible, inclusion of patient characteristics including age, gender, body mass index, diagnosis, grade of arthritis, pre-operative scores, American Society of Anaesthesiologists physical status classification, existing mental health co-morbidity such as depression and anxiety, and socioeconomic status, and procedural characteristics including prosthesis design and use of patellar resurfacing, will minimise confounding, facilitate identification of heterogeneity between studies, enable appropriate comparison of results, and aid in the translation of research findings to clinical practice settings [55,56].

### 4.2. Strengths and Limitations

This is the first review to evaluate patient-reported outcomes following primary TKR for osteoarthritis in patients <65 years of age. This cohort represents the most rapidly growing group of TKR recipients, and findings from this review may assist patients and clinicians by clarifying the outcomes that can be expected from surgery. However, the large degree of heterogeneity amongst study designs presents a source of potential bias. A wide range of instruments were reported, and inconsistencies in the reporting of raw data were frequently encountered which included missing pre-operative scores or variation in the reporting of potential confounders relating to patient or procedural characteristics. As such, only a limited meta-analysis of two prospective studies was performed, with substantial residual heterogeneity. Although large improvements following TKR are reported, it remains unclear how much of the variation in improvement following TKR is attributed to differences in patient or procedural characteristics between studies. The overall findings reflect a wide range of settings that are not directly comparable, with longer-term outcomes reflecting surgeries performed in previous decades. Findings should be cautiously interpreted with these limitations in mind.

## 5. Conclusions

The increasing use of TKR in patients <65 years of age may be supported by a large degree of clinically meaningful improvements in patient-reported pain, function, and quality of life outcomes, and the majority of these patients are satisfied with their surgery. However, results into the second postoperative decade remain uncertain, with data suggesting a high prevalence of pain and increasing functional decline. Limited evidence suggests younger patients achieved generally equivalent and potentially greater improvements in patient-reported outcomes compared to older individuals following TKR for osteoarthritis.

## Figures and Tables

**Table 1:** Patient-reported outcomes (all instruments standardised to a 0–100 scale).

Outcomes									
Study	Pre-Operative		Post-Operative		Change		Minimal Clinically Important Difference		
	Mean	SD	Mean	SD	Difference	SD	MCID (95% CI)	Method	Reference
Knee Society Score (KSS)									
McCalden et al.	N/A	N/A	N/A	N/A	78.9	N/A	N/A	N/A	N/A
Tai et al.	49.0	N/A	86.5	N/A	37.5	N/A			
KSS (Knee)									
Garabano et al.	46.8	N/A	91.4	N/A	44.6	N/A	7.2 (5.1–7.8) 7.2 (5.3–9.0)	Anchor Distribution	Lizaur-Utrilla et al., 2019 [27]
Kim et al.	25.5	N/A	94.5	N/A	69.0	N/A			
Lizaur-Utrilla et al.	34.7	11.8	87.4	8.1	52.7	N/A			
Long et al.	N/A	N/A	87.4	17.6	N/A	N/A			
Price at al.	N/A	N/A	74.8	N/A	N/A	N/A			
Tai et al.	57.0	N/A	85.0	N/A	28.0	N/A			
KSS (Function)									
Garabano et al.	49.3	N/A	92.6	N/A	43.3	N/A	9.7 (7.3–10.2) 6.3 (5.0–8.1)	Anchor Distribution	Lizaur-Utrilla et al., 2019
Kim et al.	30.0	N/A	84.5	N/A	54.5	N/A			
Lizaur-Utrilla et al.	38.1	17.0	86.3	11.4	48.2	N/A			
Long et al.	N/A	N/A	62.1	32.2	N/A	N/A			
Price et al.	N/A	N/A	56.8	N/A	N/A	N/A			
Tai et al.	43.0	N/A	90.0	N/A	47.0	N/A			
Western Ontario and McMaster Universities Osteoarthritis Index (WOMAC)									
Clement et al.	31.7	16.1	69.1	24.0	37.5	15.3	10	Anchor	Clement et al., 2018
Kim et al.	29.2	N/A	63.5	N/A	34.3	N/A			
McCalden et al.	N/A	N/A	N/A	N/A	33.3	N/A			
Townsend et al.	40.3	N/A	54.1	N/A	13.8	N/A			
WOMAC (Pain)									
Clement et al.	28.8	16.2	74.6	24.4	45.8	17.5	11	Anchor	Clement et al., 2018
Garabano et al.	N/A	N/A	88.5	N/A	N/A	N/A			
Kim et al.	45.0	N/A	88.5	N/A	43.5	N/A			
Lizaur-Utrilla et al.	39.4	6.3	88.5	4.5	49.1	N/A			
WOMAC (Function)									
Clement et al.	32.7	17.1	68.0	25.0	35.3	15.6	9	Anchor	Clement et al., 2018
Garabano et al.	N/A	N/A	59.9	N/A	N/A	N/A			
Kim et al.	24.3	N/A	56.0	N/A	31.7	N/A			
Lizaur-Utrilla et al.	49.2	8.1	83.9	6.6	34.7	N/A			
WOMAC (Stiffness)									
Clement et al.	30.0	20.6	65.5	24.8	35.2	19.1	8	Anchor	Clement et al., 2018
Garabano et al.	N/A	N/A	67.5	N/A	N/A	N/A			
Kim et al.	31.9	N/A	65.6	N/A	33.7	N/A			
Oxford Knee Score (OKS)									
Niemeläinen et al.	45.8	16.0	85.4	16.0	39.6	N/A	19.2	Anchor	Beard et al., 2015 [28]
Price at al.	N/A	N/A	64.4	N/A	N/A	N/A			
Townsend et. al.	30.0	N/A	44.4	N/A	14.4	N/A			
OKS (Pain)									
Townsend et al.	25.6	N/A	40.1	N/A	14.5	N/A	25 (22–27.5)	Anchor	Clement et al., 2014 [29]
OKS (Function)									
Townsend et al.	32.4	N/A	47.5	N/A	15.2	N/A	15.4 (13.6–17.1)	Anchor	Clement et al., 2014 [30]
Hospital for Special Surgery Knee Score (HSS)									
Kim et al.	48.0	N/A	91.5	N/A	43.5	N/A	N/A	N/A	N/A
Long et al.	57.9	10.3	85.3	13.2	27.4	N/A			
High Activity Arthroplasty Score (HAAS)									
Niemeläinen et al.	33.3	21.1	61.1	21.1	27.8	N/A	N/A	N/A	N/A
Knee Injury and Osteoarthritis Outcome Score (KOOS) Pain									
Niemeläinen et al.	45.0	15.4	86.0	15.4	41.0	N/A	16.7	Anchor	Monticone et al., 2013 [31]
Knee Injury and Osteoarthritis Outcome Score (KOOS) Symptoms									
Niemeläinen et al.	43.0	19.2	79.0	19.2	36.0	N/A	10.7	Anchor	Monticone et al., 2013
Knee Injury and Osteoarthritis Outcome Score (KOOS) QOL									
Niemeläinen et al.	21.0	15.4	70.0	23.1	49.0	N/A	15.6	Anchor	Monticone et al., 2013
Knee Injury and Osteoarthritis Outcome Score (KOOS) ADLs function									
Niemeläinen et al.	50.0	15.4	88.0	15.4	38.0	N/A	18.4	Anchor	Monticone et al., 2013
Knee Injury and Osteoarthritis Outcome Score (KOOS) Sport/recreation function									
Niemeläinen et al.	14.0	19.2	55.0	30.7	41.0	N/A	12.5	Anchor	Monticone et al., 2013
Short Form-12 Physical Component Score (SF-12 PCS)									
Clement et al.	26.9	7.0	38.1	12.2	11.2	7.8	1.8 (0.1–3.5)	Anchor	Clement et al., 2019 [32]
Lizaur-Utrilla et al.	20.1	6.0	82.6	6.9	N/A	N/A			
Short Form-12 Mental Component Score (SF-12 MCS)									
Clement et al.	41.6	13.9	44.5	14.7	2.9	9.4	Nil significant	Anchor	Clement et al., 2019
Lizaur-Utrilla et al.	47.2	10.4	74.6	8.1	N/A	N/A			
Research and Development-36 Physical Component Score (RAND-36 PCS)									
Niemeläinen et al.	37.0	19.2	68.0	23.1	31.0	N/A	N/A	N/A	N/A
Research and Development-36 Mental Component Score (RAND-36 MCS)									
Niemeläinen et al.	61.0	23.1	79.0	15.4	18.0	N/A	N/A	N/A	N/A

**Table 2:** Study characteristics.

Study Characteristics															
Study	Design	TKRs	Prosthesis	Patellar Resurfacing	Age	Osteoarthritis (%)	Surgery Period	Follow-Up (Range)	Follow-Up %	Disease-Specific Instruments	Generic Health Instruments	Satisfaction Instruments	Difference in Outcomes Compared to Older Patients	Summary of Statistical Significance	Summary of Clinical Significance
Clement et al., 2018	Retrospective	224	N/A	N/A	<55	100	2003–2013	1 year	N/A	WOMAC	SF-12 (PCS & MCS)	Four-point Likert scale	WOMAC total: −0.8 (*p* = 0.57) WOMAC pain: −1.2 (*p* = 0.46) WOMAC function: −1.6 (*p* = 0.29) SF-12 PCS: 1.3 (*p* = 0.06) SF-12 MCS: 0.8 (*p* = 0.37) Satisfaction: 83.4% (<55 years) vs. 92.0% (≥55 years) (*p* = 0.001) no statistical difference for above scores following regression adjustment for patient demographics and pre-operative scores	WOMAC total: equivalent WOMAC pain: equivalent WOMAC function: equivalent SF-12 PCS: equivalent SF-12 MCS: equivalent Satisfaction: inferior satisfaction considered equivalent by authors after attribution to higher prevalence of mental health disorders in younger age group	No difference as did not exceed MCID
Niemeläinen et al., 2018	Prospective	227	CR 96%, PS 4%	12/227	Mean 58 (<65)	100	2012–2014	2 years (22 to 26 months)	93%	OKS, HAAS, KOOS	RAND-36 (PCS & MCS)	100-point Visual Analogue Scale	N/A	N/A	N/A
Lizaur-Utrilla et al., 2017	Prospective	61	CR 100%	61/61	Median 53 (30–55)	100	2001–2005	12 (10 to 14) years	100%	KSS, WOMAC	SF-12 (PCS & MCS)	Satisfaction assessed but instrument not specified	Five year follow-up KSS knee: −0.6 (*p* > 0.05) KSS function: −0.2 (*p* > 0.05) WOMAC pain: −3.3 (*p* > 0.05) WOMAC function: 4.0 (*p* = 0.001) SF-12 PCS: 0.5 (*p* > 0.05) SF-12 MCS: −0.6 (*p* > 0.05) Latest follow-up KSS knee: 2.5 (*p* > 0.05) KSS function: 5.2 (*p* = 0.018) WOMAC pain: −3.1 (*p* > 0.05) WOMAC function: 2.7 (*p* = 0.028) SF-12 PCS: 2.4 (*p* = 0.001) SF-12 MCS: 2.4 (*p* = 0.035) Satisfaction: 90.2% (<55 years) vs. 87.9% (≥55 years) (*p* > 0.05)	Five year follow-up KSS knee: equivalent KSS function: equivalent WOMAC pain: equivalent WOMAC function: superior SF-12 PCS: equivalent SF-12 MCS: equivalent Latest follow-up KSS knee: equivalent KSS function: superior WOMAC pain: equivalent WOMAC function: superior SF-12 PCS: superior SF-12 MCS: superior Satisfaction: equivalent	Difference in KSS function within 95% CI of MCID by distribution method Difference in SF-12 PCS and MCS within 95% CI of MCID by anchor method
Townsend et. al., 2017	Retrospective	100	N/A	N/A	<60	100	2008–2014	6 months	N/A	WOMAC, OKS	N/A	N/A	WOMAC total: −3.3 (*p* = 0.0007) OKS total: −0.8 (*p* < 0.0001) outcomes are weighted by sample size comparing patients <60 years vs. patients ≥70 years; one subgroup was age 60–69 years which was excluded from comparison, *p* values are after adjustment for baseline scores by authors	WOMAC total: inferior OKS total: inferior	No difference as did not exceed MCID
Garabano et al., 2016	Retrospective	53	PS 100%	40/53	Mean 49 (26–54)	96	1997–2011	6.5 (2 to 15) years	N/A	KSS, WOMAC	N/A	Satisfaction assessed but instrument not specified	N/A	N/A	N/A
Long et al., 2014	Prospective	38	PS 100%	N/A	Mean 51 (22–55)	100	1977–1992	25.1 (20 to 35) years	95%	KSS, HSS	N/A	N/A	N/A	N/A	N/A
McCalden et al., 2013	Retrospective	673	CR 12%, PS 88%	660/673	Mean 50 (<55)	90	1996–2009	2+ years	N/A	KSS, WOMAC	SF-12 (PCS & MCS)	N/A	KSS total: 9.9 (*p* < 0.001) KSS knee: greater improvement but value N/A (*p* < 0.001) KSS function: greater improvement but value N/A (*p* < 0.001) WOMAC total: 6.0 (*p* < 0.001) WOMAC pain: greater improvement but value N/A (*p* < 0.001) WOMAC function: greater improvement but value N/A (*p* < 0.001) WOMAC stiffness: greater improvement but value N/A (*p* < 0.001) SF-12 PCS: no significant difference (*p* > 0.05) SF-12 MCS: no significant difference (*p* > 0.05) comparing patients ≤55 years vs. patients >70 years; one subgroup was age 55–70 years which was excluded from comparison	KSS total: superior KSS knee: superior KSS function: superior WOMAC total: superior WOMAC pain: superior WOMAC function: superior WOMAC stiffness: superior SF-12 PCS: equivalent SF-12 MCS: equivalent	Values or MCID unavailable to enable determination
Kim et al., 2012	Prospective	216	CR 50%, PS 50%	N/A	Mean 45 (<51)	100	1993–1996	16.8 (15 to 18) years	95%	KSS, WOMAC, HSS	N/A	N/A	N/A	N/A	N/A
Price at al., 2010	Prospective	37	CR 100%	5/37	Mean 55.4 (32–59.5)	100	1987–1993	15.7 (12 to 19) years	60%	KSS, OKS	N/A	N/A	N/A	N/A	N/A
Tai et al., 2006	Prospective	118	CR 100%	43/118	Mean 50.7 (32–55)	100	1992–2000	7.9 (5 to 12.5) years	99%	KSS	N/A	N/A	N/A	N/A	N/A

**Table 3:** Satisfaction.

Satisfaction						
Authors	TKRs	Satisfaction	Domain	Instrument	Criteria	Follow-Up
Clement et al.	224	83.4% 85.1% 80.1% 72.4%	Overall Pain Work Recreation	Four-point Likert scale: Very satisfied Somewhat satisfied Very dissatisfied Somewhat dissatisfied	Very satisfied Somewhat satisfied	1 year
Niemeläinen et al.	227	81% at 1 year 84% at 2 years	Overall	Visual Analogue Scale: Very satisfied (76–100 points) Satisfied (51–75 points) Unsure (26 to 50 points) Dissatisfied (0–25 points)	Very satisfied (76–100 points) Satisfied (51–75 points)	1 year 2 years
Garabano et al.	53	93.5%	Overall	N/A	N/A	6.5 years
Lizaur-Utrilla et al.	61	90.2%	Overall	N/A	N/A	10 years

**Table 4:** Assessment of methodological quality.

Study	Confounding	Selection of Participants	Classification of Interventions	Missing Data	Outcome Measurement	Selective Reporting	Overall Risk of Bias
Clement et al., 2018							
Niemeläinen et al., 2018							
Lizaur-Utrilla et al., 2017							
Townsend et. al., 2017							
Garabano et al., 2016							
Long et al., 2014							
McCalden et al., 2013							
Kim et al., 2012							
Price at al., 2010							
Tai et al., 2006							

Green—low risk of bias, yellow—moderate risk of bias, red—high risk of bias.
